# Country Differences in Fall Risk Among Older Adults: A Comparative Study of India and the USA

**DOI:** 10.1093/geroni/igaf122.2149

**Published:** 2025-12-31

**Authors:** Shekhar Chauhan, Dawn Carr

**Affiliations:** Florida State University, Tallahassee, Florida, United States; Florida State University, Tallahassee, Florida, United States

## Abstract

Falls represent a critical health concern for older adults, with risk factors varying across socio-demographic and lifestyle contexts. Despite significant implications for public health, few studies have compared fall risk between older adults in different countries. This study examines how socio-economic status and socio-demographic factors influence fall risk among older adults (age 65+) in the U.S and India. Using a harmonized cross-sectional dataset we created using data drawn from the Longitudinal Ageing Study in India (LASI 2017-18) and the Health and Retirement Study (HRS, 2016 & 2018), we used logistic regression models to identify factors that predict fall risk, and interaction tests to evaluate if fall-related risk factors are moderated by country (i.e., USA vs. India). Results show that older adults in the USA have almost three times the odds of experiencing a fall compared to their counterparts in India (p < 0.001). Regarding moderators, we find that more years of education was associated with higher fall risk in the US (p < 0.001) (but not in India), and females have elevated risk of Falls in India (p < 0.001), but not in the US relative to their male counterparts. Although rural-living older adults face elevated fall risk in both countries, rural-living older adults in the US adults face particularly high risk of falling. We conclude with a discussion about implications for country-specific fall prevention strategies.

